# Examination for Degrees

**Published:** 1866-02

**Authors:** 


### EXAMINATIONS FOR DEGREES.

We do not know what methods are adopted by our dental schools in conducting their "final examinations." The Ohio College has varied. The recent examinations were conducted in writing, and lasted, with each professor, from one to two and a half hours. The questions, or propositions, were written on the blackboard, or furnished on slips, and the class sat in their seats, as usual, and wrote out their answers. The faculty met, agreed on a per cent of correct answers requisite to graduation; and then compared notes, enabling the whole faculty to vote intelligently as to the correctness or incorrectness of each answer. The simple summing up of the tally-sheet decided the fate of the candidate.

The older alumni of the institution will see by this that the examination has been much more thorough than in their day. By this mode, each student is subjected to an examination of eight or nine hours. He, however, avoids the embarrassment of a new position. He writes his answers, as he usually sits, in the familiar lecture room, and in the presence of but one professor at a time. In this respect, a fairer test of attainment is afforded than can be obtained in the embarrassments incident to the "green-room." While rigid and thorough, it is fair and satisfactory. W.

